# Microhemorrhage in a Rat Model of Neonatal Shaking Brain Injury: Correlation between MRI and Iron Histochemistry

**DOI:** 10.1267/ahc.20007

**Published:** 2020-08-06

**Authors:** Daisuke Taguchi, Ayuka Ehara, Yoshiteru Seo, Shuichi Ueda

**Affiliations:** 1 Department of Judo Therapy, Faculty of Medical Technology, Teikyo University, Utsunomiya, Tochigi 320–8551, Japan; 2 Department Histology and Neurobiology, Dokkyo Medical University, School of Medicine, Shimotsuga, Tochigi 321–0293, Japan; 3 Department of Regulatory Physiology, Dokkyo Medical University, School of Medicine, Shimotsuga, Tochigi 321–0293, Japan

**Keywords:** rat model, shaking brain injury, magnetic resonance imaging, iron histochemistry

## Abstract

Previous studies have shown that neonatal shaking brain injury (SBI) causes transient microhemorrhages (MHs) in the gray matter of the cerebral cortex and hippocampus. Iron deposits and iron-uptake cells are observed surrounding MHs in this SBI model, suggesting local hypoxic-ischemic conditions. However, whether the shaken pups suffered systemic hypoxic-ischemic conditions has remained uncertain. Further, histopathological correlations of MHs on magnetic resonance imaging (MRI) are still unclear. The present study examined MHs after neonatal SBI using a combination of histochemical and susceptibility-weighted imaging (SWI) analyses. Systemic oxygen saturation analyses indicated no significant difference between shaken and non-shaken pups. MHs on postnatal day 4 (P4) pups showed decreased signal intensity on SWI. Iron histochemistry revealed that these hypointense areas almost completely comprised red blood cells (RBCs). MHs that appeared on P4 gradually disappeared by P7–12 on SWI. These resolved areas contained small numbers of RBCs, numerous iron-positive cells, and punctate regions with iron reaction products. Perivascular iron products were evident after P12. These changes progressed faster in the hippocampus than in cortical areas. These changes in MHs following neonatal SBI may provide new insights into microvascular pathologies and impacts on brain functions as adults.

## I Introduction

Using susceptibility-weighted imaging (SWI), we have demonstrated that repeated mild shaking brain injury (SBI) in neonatal rat pups induces transient microhemorrhages (MHs) in the gray matter, especially in the frontal and parietal cerebral cortices and hippocampus [11, 23]. Furthermore, sensitive iron staining on histochemical examination demonstrated leakage of iron and iron-positive cells in the brain, with these staining patterns continuing after disappearance of hemorrhagic signals on SWI [11]. Iron is an important cofactor involved in a variety of body functions. In the brain, intracellular iron is normally sequestered within ferritin, which stabilizes the highly reactive iron [7]. Since iron is a transition metal, Fe^2+^ and Fe^3+^ can react with hydrogen peroxide and oxygen, respectively, leading to the formation of reactive oxygen species (ROS) via the Fenton reaction and Haber-Weiss cycle [3, 9]. The neuropathological roles of abnormally accumulated iron have been reported in hemorrhagic brain disease, such as subarachnoid hemorrhage, intracerebral hemorrhage and traumatic brain injury [13]. Together, our data suggest the presence of iron-associated gray matter injury after MHs. However, the role of iron in the pathogenesis of this model remains under investigation. Although iron-staining patterns were classified into three types according to morphology [11], detailed analyses regarding the temporal progression of MHs and correlations with findings from SWI have remained lacking.

A previous rat model of neonatal SBI has shown that the procedure of shaking alone is insufficient to produce intracerebral hemorrhage (ICH), but the procedure of shaking combined with hypoxia leads to ICH [22]. Thus, for the present study, we measured systemic oxygen saturation (SaO_2_) to ensure hypoxic conditions in our model. We then examined correlations between MHs on SWI and iron-staining patterns. In SBI models, MH-induced delayed white matter damage has usually been identified in the corpus callosum (CC) [1, 5]. Finally, to determine whether evident demyelination has occurred, we measured CC thickness in this SBI model compared to adult non-shaking control rats.

## II Materials and Methods

### Animals and experimental design

All experimental procedures received prior approval by the Animal Welfare Committee of Dokkyo Medical University School of Medicine, and were conducted in accordance with the National Institutes of Health Guide for the Care and Use of Laboratory Animals.

Pregnant female Sprague-Dawley rats were purchased from Charles River Laboratories (Tsukuba, Japan) and housed under conditions of controlled temperature (22 ± 2°C) and humidity (50–60%) and a regular 12-hr light-dark cycle with ad libitum access to food and water. Date of birth was considered as postnatal day 0 (P0). The shaking apparatus and procedures were based on our previous paper [11]. Briefly, male rat pups were anesthetized with isoflurane and shaken for 60 sec, then rested for 60 sec, with this process repeated 5 times/day for the 5 days from P3 to P7 (S group). Controls (C group) only underwent placement in the chamber of the shaking apparatus without shaking during the same experimental period under the same conditions of anesthesia. After the experiment, all pups were returned to the dam. At P21, offspring were weaned and placed in a cage (2–3 rats per cage) until the experiment.

### SaO_2_ measurement

SaO_2_ was measured by pulse oximetry in connection with a monitoring and gating system (Model 1025T; SAI, Stony Brook, NY). Following the shaking of P6 pups from S group, a sensor clip (PART #531100; SAI) was immediately (within 2 min) attached to a hindlimb, then SaO_2_ was measured continuously for 15 min under anesthesia. Data were compared to pups of the same age from C group and a group shaken continuously for 10 min (continuous shaking group) under the same conditions of anesthesia.

### Tissue sample preparation

Under deep anesthesia with pentobarbital, 2 hr after shaking on P3, P4, P7, P12, P14, P35 and P70, S group rats and age-matched C group rats (n = 4 each) were transcardially perfused with physiological saline, followed by 4% paraformaldehyde in 0.1-M phosphate buffer (PB, pH 7.4) at 4°C. The brain was removed from the skull and placed in the same fixative for 3–5 days at 4°C, then immersed in 30% sucrose in 0.1-M PB. To protect tissues from damage during freezing and sectioning, the brains of P3–12 rats were rapidly embedded with 10% gelatin and further fixed into the same fixative [24]. The sample was then placed in embedding compound and frozen in powdered dry ice. Serial frontal sections (thickness, 40 μm) were made using a cryostat (HM525NX; Thermo Fisher Scientific, Runcorn, UK). Sections were serially divided into 6 groups and placed in 0.1-M phosphate-buffered saline (PBS, pH 7.4).

### Histochemical and immunohistochemical staining

To identify the location of MHs, one series of sections was incubated with 0.5% 3,3'-diaminobenzidine (DAB) in PB for 15 min, then 600 μL of 30% hydrogen peroxide was then added for every 20 mL of DAB solution to visualize the endogenous peroxidase of red blood cells (RBCs). Some sections were counterstained with 1% methyl green. Using these sections, the diameter of MHs in P3 cortex and hippocampus and distance from the pial surface to the MH core in P3 and P7 rat cortex were measured.

For iron staining, the next two series of sections were treated with and without 1% H_2_O_2_ in 0.1-M PB at room temperature for 1 hr (to quench endogenous peroxidase activity), then a modified Perls’ method was used for iron staining [8]. Sections were then washed in 0.1-M PBS, mounted on slides, dehydrated and cover-slipped. To identify the glial cell types of iron-positive cells, double-labeling with iron staining and immunostaining for Iba1 or glial fibrillary acidic protein (GFAP) was performed. Briefly, sections were washed with 0.3% Triton X in 0.05-M Tris-buffered saline (TBS; pH 7.6), then with 10% normal goat serum and 0.1% bovine serum albumin in TBS to block non-specific reactions. Sections were incubated with rabbit anti-Iba1 antibody (dilution 1:2,000; FUJIFILM Wako Pure Chemical Corporation, Osaka, Japan) or mouse anti-GFAP antibody (dilution 1:5,000; Sigma Aldrich, Saint Louis, MO) at 4°C overnight. After rinsing 3 times in TBS, sections were incubated with alkaline phosphatase-conjugated goat anti-rabbit or anti-mouse immunoglobulin G secondary antibody (dilution 1:500; Sigma Aldrich) for 60 min at room temperature. After rinsing 3 times in TBS, sections were incubated for 5 min in 0.1-M Tris HCl (pH 8.2) containing Vector Blue substrate (Vector Lab, Burlingame, CA) and 0.98% levamisole, then a modified Perls’ method was treated. Sections cleaned with Histo-Clear (National Diagnostics, Atlanta, GA) were mounted in VectaMount (Vector Lab).

To clarify the locations of RBCs and cerebral blood vessels, another series of sections was immunohistochemically stained for rat endothelial cell antigen 1 (RECA-1) to identify blood vessels and histochemically for endogenous peroxidase staining to identify RBCs [11]. Briefly, for double-labeling, sections were pretreated with normal goat serum in TBS to block non-specific reactions. Sections were incubated with mouse anti-rat RECA-1 antibody (dilution 1:20,000; SEROTEC, Oxford, UK), then incubated with alkaline phosphatase-conjugated goat anti-mouse immunoglobulin G secondary antibody. After developing with Vector Blue substrate in 0.1-M Tris HCl (pH 8.2), sections were incubated with DAB in Tris HCl (pH 7.6) and H_2_O_2_ was added to visualize RBCs. Stained sections were mounted in Immu-Mount (Thermo Scientific, Rockford, PA).

### MRI

**Fig. 1.**
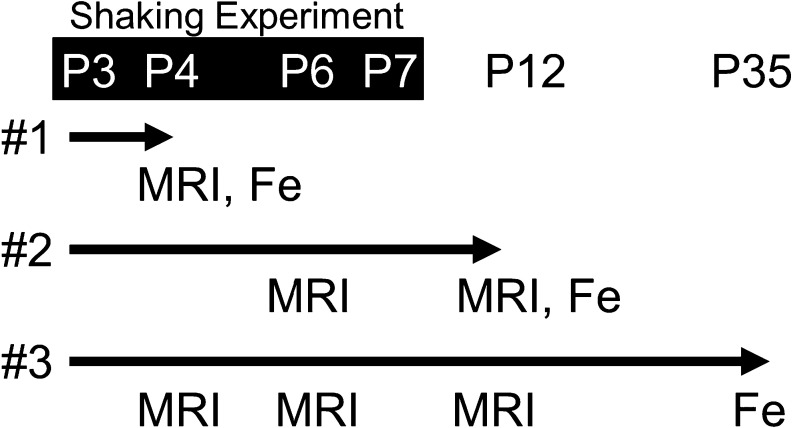
MRI and iron-staining (Fe) schedule for S group rats. P3–7 rat pups were shaken according to protocol 5 times per day (white letters on black background). These rats were divided into three experimental groups (#1–3): #1 group (n = 4), perfused for iron-staining immediately after MRI on P4; #2 group (n = 2), analyzed by MRI on P6 and P12, then perfused for iron-staining immediately after MRI on P12; and #3 group (n = 5), analyzed by MRI on P4, P6 and P12, then perfused for iron-staining on P35.

To investigate MHs and edematous changes in living animals (P4, P6 and P12 of S group; n = 11), we used SWI from magnetic resonance imaging (MRI). Details of the methods and imaging conditions have already been reported in our previous paper [11]. During MRI measurement, each pup was anesthetized with 1% sevoflurane in a gas mixture of O_2_/CO_2_/N_2_O delivered through a facemask at 0.4 L min^−1^. Body temperature was monitored using a fluorescence thermometer (AMOS FX-8000-210; Anritsu Meter, Tokyo, Japan). Using a high-field MRI system (7-T AVANCE III; Bruker Biospin, Ettlingen, Germany), even small amounts of deoxyhemoglobin can be detected as signal hypointensity compared with surrounding intact tissues. We can thus detect small hemorrhagic legions. Original images were detected using an 18-mm surface coil (Doty Scientific, Columbia, SC), and by T_1_-weighted gradient-echo imaging using the following settings: field of view, 19.2 × 19.2 mm; data matrix, 256 × 256; slice thickness, 0.5 mm; 7 slices with 0.7-mm interval; repetition time, 250 ms; echo time, 15 ms; and flip angle, 45°. SWI processing was applied with a mask weighting value of 3 and Gaussian broadening of 1 mm. Pups were returned to the dam and spots of MH were traced for several days. Figure 1 indicates the experimental schedules for SWI and iron histochemistry. After SWI, P4 (Fig. 1 #1, n = 4), P6 (Fig. 1 #2, n = 2) and P12 (Fig. 1 #3, n = 5) rats underwent immediate perfusion with fixative to examine correlations between SWI and iron histochemistry.

### Changes in MHs after shaking and analysis of white matter thickness

In our previous paper [11], the histochemical pattern of staining for iron in this model was classified into three types: hemorrhage (H) type; mixed (M) type showing RBCs, iron-positive cells and punctate iron reaction products; and perivascular (P) type consisting of enlarged vessels, iron-positive cells and punctate iron reaction products. Percentages of each iron-staining pattern were calculated in the cortex and hippocampus of P3 (n = 8), P7 (n = 7), P14 (n = 4), and P35 (n = 5) shaken rats.

**Fig. 2.**
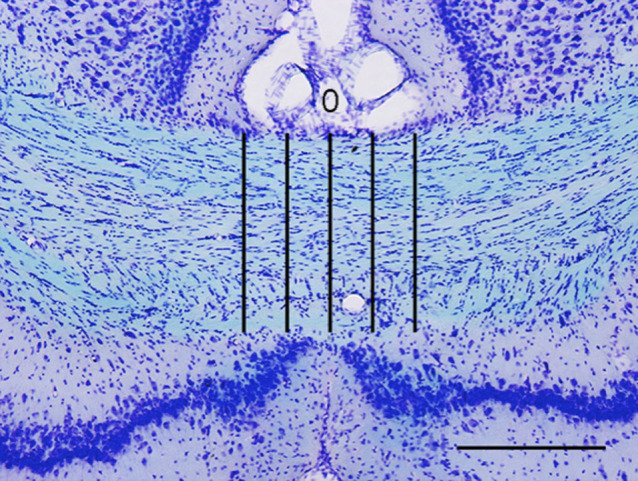
Representative image of an LFB- and CV-stained section. Vertical line through the center of the CC is defined as the 0 line, and thickness is measured based on this line. Four thickness measurements are taken from lines parallel to the 0 line (± 100 μm and ± 200 μm from 0). Bar = 300 μm.

To evaluate white matter damage associated with MHs after SBI [5], the body of the CC at the level of the bregma −3.32 mm [18] was selected for white matter analysis in P70 rats from S group (n = 5) and age-matched rats from C group (n = 5). Typical sections from samples were mounted on slides, then stained with Luxol fast blue (LFB) and cresyl violet (CV). Briefly, slides were washed in 95% ethanol and incubated with 0.1% LFB overnight at 60°C. Slides were then rinsed in 95% ethanol and further washed under tap water to remove excess stain. Stain differentiation was carried out in 0.5% lithium carbonate solution. Slides were lightly counterstained in 0.1% CV for 3–10 min at 37°C, dehydrated, cleared in xylene, and coverslipped with Entellan New (MERCK, Darmstadt, Germany). Quantitative measurement of white matter thickness was based on the methods described by Brooks *et al.* [2]. A vertical line through the center of the CC was defined as the 0 line and CC thickness was measured. Another four thickness measurements were taken from lines parallel to the 0 line (± 100 μm and ± 200 μm from 0) (Fig. 2). Mean thickness was calculated for each rat.

### Statistics

Data are presented as mean ± standard error of the mean. Statistical measurements were performed using Stat-View (Abacus Concepts, Berkeley, CA). For oxygen saturation, two-factorial (way) analysis of variance (ANOVA) was performed with group and time as the main factors. Student’s t-test was used for comparison of diameters, distance from the pial surface to MHs, and CC thickness. Changes were identified as significant for values of p < 0.05.

## III Results

### SaO_2_ during shaking

**Fig. 3.**
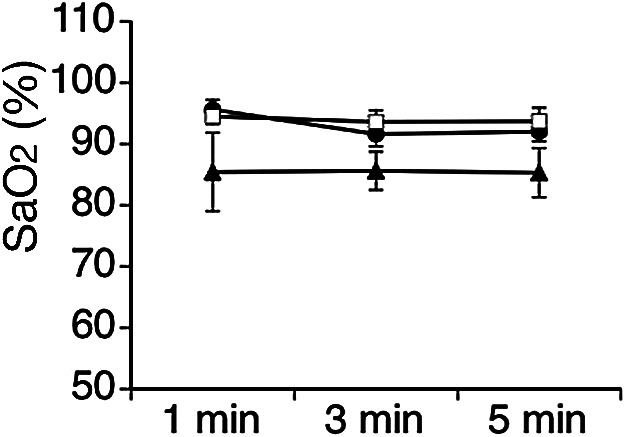
Time courses for arterial oxygen saturation (SaO_2_) in C group (black circles), S group (white squares), and 10-min continuous shaking group (black triangles) after shaking. No significant differences are seen between groups.

In peripheral SaO_2_ analyses, continuously shaken P6 rats showed lower SaO_2_ than S group and C group P6 rats (Fig. 3). However, two-factor ANOVA showed no significant group effect (F(2,10) = 2.16, p = 0.16), time effect (F(2,10) = 0.86, p = 0.43) or group × time effect (F(4,20) = 0.56, p = 0.68).

### Distribution of MHs and abnormal iron deposition in P3–P7 rats

**Fig. 4.**
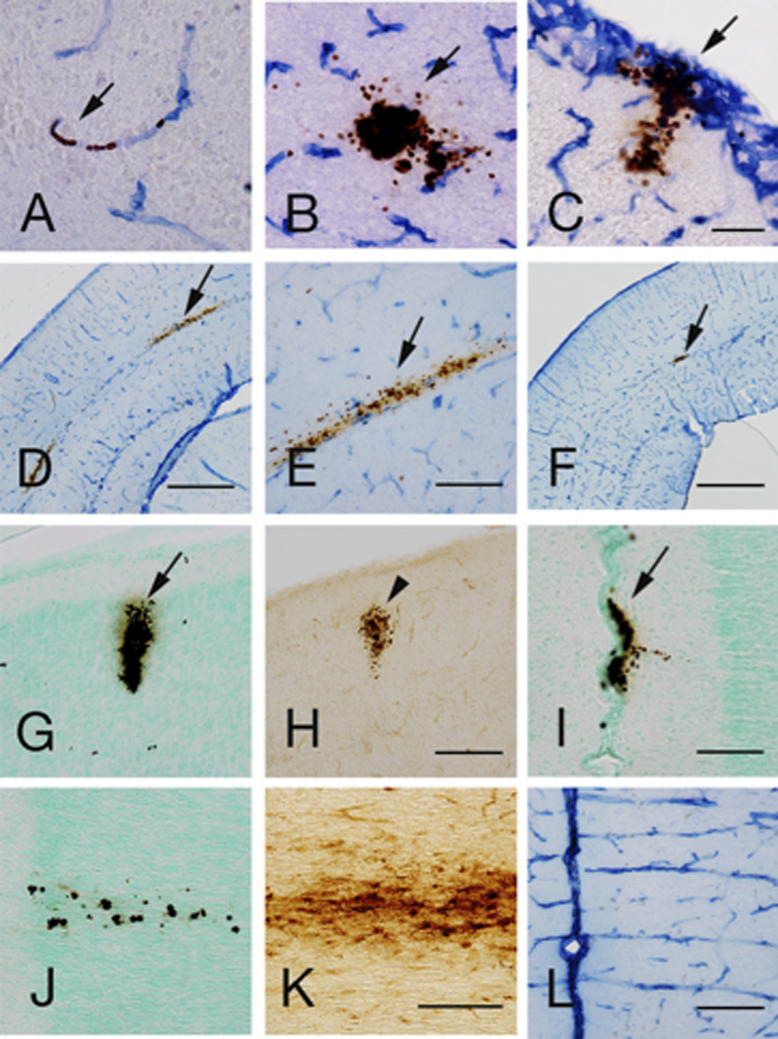
Histochemical and/or immunohistochemical staining of coronal sections though the parietal cortex on P3 (**A–C**) and P7 (**G, H**), visual cortex on P3 (**D, E**) and P7 (**F**), and medial prefrontal cortex on P7 (**I–L**) for shaken rats (**B–K**) and non-shaken rats (**A, L**). **A–F**: Double-labeling of blood vessels and RBCs in the cortical parenchyma (**A–B**), cortical surface (**C**) and deep white matter between lateral external capsule and hippocampal alveus (**D–F**). **G–K**: DAB histochemical staining and counterstaining with methyl green (**G, I, J**) and iron-histochemical staining without H_2_O_2_ pretreatment (**H, K**). In non-shaken rats, DAB-stained RBCs are located within blood vessels (arrow; **A**), whereas extravasation of RBCs is observed in shaken rats (arrows; **B–G, I**). MHs in the leptomeningeal plexus are evident (arrows; **C, I**). MHs mainly comprise RBCs, representing H-type staining (**G, H**). In the mPFC, DAB-stained RBCs are sparsely distributed along penetrating vessels (**J**). An adjacent section stained for iron histochemistry shows iron-positive cells and punctate iron reactions in columnar distributions (**K**). Endothelial cells are stained by RECA-1 immunohistochemistry. Penetrating blood vessels in the cerebral cortex are evident (**L**). Bar = 50 μm (**A–C**), 500 μm (**D**), 200 μm (**E, G, H, L**), 1 mm (**F**), 100 μm (**I, J, K**).

In non-shaken (C group) rats with double-immunohistochemical staining, RBCs were demonstrated within RECA-1-positive capillary vessels (Fig. 4A), while extravasation of RBCs was usually observed in the gray matter of the cerebral cortex and hippocampus of S group rats, as described previously [11, 23] (Fig. 4B). The present study further found MHs in the brain surface of P3 cerebral cortex that continued to the leptomeningeal plexus (Fig. 4C, I). Furthermore, MHs aligned with deep white matter between the lateral external capsule and hippocampal alveus were occasionally observed in S group rats at P3–7 (Fig. 4D-F). As described previously [11, 23], H-type iron staining mainly comprising RBCs was observed in the cerebral cortex of P3–7 rats from S group (Fig. 4G, H). In the medial prefrontal cortex (mPFC) of S group at P3–7, DAB-positive RBCs were occasionally observed in the gray matter, arranged vertically to the cortical surface (Fig. 4J). Adjacent sections stained for iron histochemistry revealed the presence of many iron-positive cells and punctate iron reactions in this area (Fig. 4K). RECA-1 immunohistochemical staining of P7 C group rats showed vessels penetrating in the mPFC (Fig. 4L). The mean diameter of MHs was significantly higher in cerebral cortex (120.3 ± 21.8 μm) than in hippocampus (41.0 ± 7.7 μm, p < 0.05).

### Morphological correlation between images from SWI and iron-staining patterns in S group, and temporal changes to iron-staining pattern in S group

**Fig. 5.**
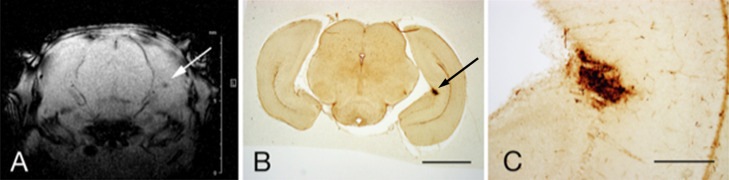
**A**: Representative in-vivo coronal-section SWI through the hippocampus of an S group rat at P4. **B**: Corresponding section stained for iron histochemistry. Arrows in **A** and **B** indicate microhemorrhages. **C**: High magnification view of the focal hemorrhage in **B**. Bar = 1 mm (**B**), 300 μm (**C**).

**Fig. 6.**
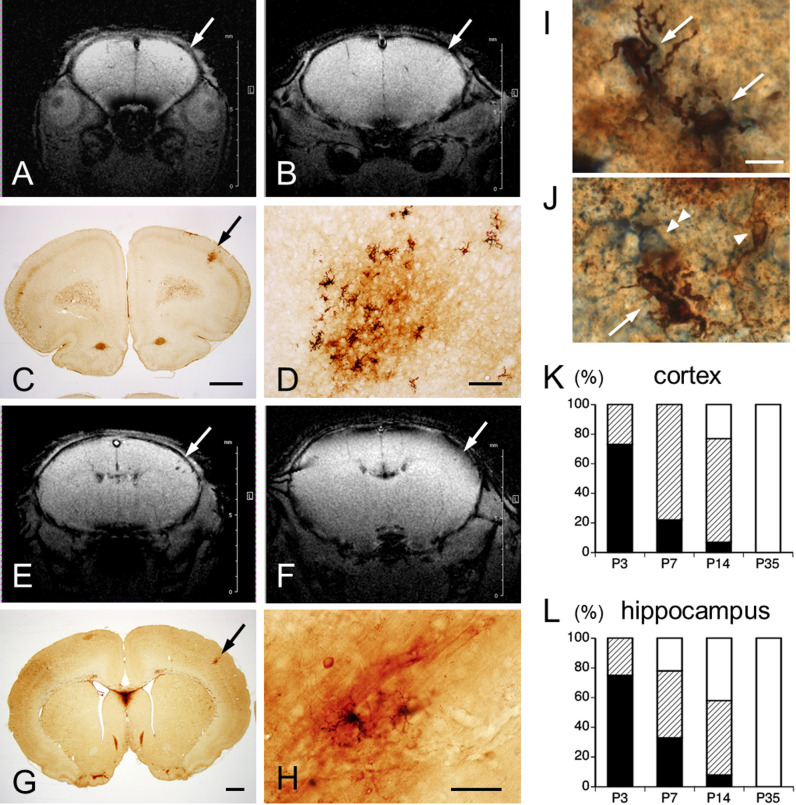
**A, B, E, F**: Representative coronal section *in vivo* SWI through the primary motor area (**A, B**) and primary sensory area (**E, F**) of the same S group rats on P6 (**A, E**) and P12 (**B, F**). Arrows indicate hemorrhagic area at which hypointense signals gradually diminish on P12 (**B, F**). Images **B** and **F** are images from SWI corresponding to the iron histochemistry-stained sections shown in **C**, **G**, respectively. **D, H**: High-magnification views of M- and P-type staining in **C** and **G** (arrows), respectively. Double-labeled cells (arrows) with iron (arrowhead) and Iba1 (**I**), or GFAP (**J**, double arrowhead). Bar = 1 mm (**C, G**), 100 μm (**D, H**), 10 μm (**I, J**). **K, L**: Quantification of MH types in cerebral cortex (**K**) and hippocampus (**L**) of P3, P7, P14, and P35 rats. Percentages staining pattern types for iron histochemistry: H-type, black column; M-type, hatched column; P-type, white column.

As described previously [11], cortical and hippocampal MHs of P4 rats showed decreased signal intensity areas on SWI (Fig. 5A). These hypointense areas consisted almost entirely of RBCs (Fig. 5B, C). In the cerebral cortex, this appearance in SWI continued at P6 (Fig. 6A, E). At P12, these MHs were seen to be gradually disappearing (Fig. 6B, F). Iron histochemical staining of the same rats showed vestigial MHs, consisting of RBCs, iron-positive cells and punctate areas of iron reaction products (Fig. 6C, D, G, H). Since iron-positive cells were morphologically similar to microglia or astrocytes, double-labeling with Perls’ method and each glial cell marker was performed to confirm cell type. Most iron-positive cells showed no labeling with glial markers, but some cells were immunopositive for microglial marker Iba1 (Fig. 6I). Although rare, a small number of cells were immunopositive for the astrocyte marker GFAP (Fig. 6J).

**Fig. 7.**
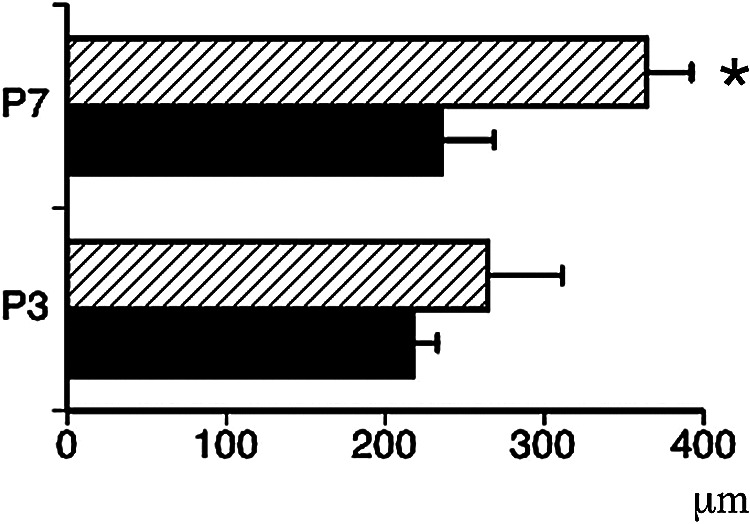
Distance from pial surface to MH core in S group rats on P3 and P7. H-type, black column; M-type, hatched column. * p < 0.05 versus H type at P7.

Percentages of iron-staining patterns in the cerebral cortex and hippocampus are indicated in Figure 6K and 6L, respectively. Percentage iron-staining patterns in the cerebral cortex were as follows: P3-H type (73%), M type (27%) and P type (0%); P7-H type (22%), M type (78%) and P type (0%); P14-H type (7%), M type (70%) and P type (23%); and P35-P type (100%). Percentages of iron-staining patterns in the hippocampus were as follows: P3-H type (75%), M type (25%) and P type (0%); P7-H type (33%), M type (45%) and P type (22%); P14-H type (8%), M type (50%) and P type (42%); and P35-P type (100%). Distance from the pial surface to MH core on P7 was significantly deeper in M type (364.2 ± 28.2 μm) than in H type (234 ± 33.5 μm; p < 0.01), whereas no significant difference was seen on P3 between M type (264.2 ± 47.0 μm) and H type (217.4 ± 15.3 μm; p = 0.22) (Fig. 7). No significant differences were identified between H type on P3 and P7 (p = 0.61), or between M type on P3 and P7 (p = 0.09).

### White matter thickness of the CC

In analyses of CC thickness, no significant differences were found between S group (461.2 ± 16.9 μm) and C group (490.0 ± 22.0 μm) rats (p = 0.33).

## IV Discussion

The present study monitored peripheral SaO_2_ during 5 min following SBI in addition to observation of skin color to evaluate systemic hypoxic conditions. In our experiments with neonatal SBI, no significant differences in peripheral SaO_2_ or skin color changes were evident between S group P6 rats and naïve (non-shaken) P6 rats. These data support the absence of any systemic hypoxic changes in the present SBI model.

Shaking results in mechanical-type injury in which the brain shifts mainly under linear translation with some additional rotation, providing inertial and/or angular acceleration and deceleration forces, causing straining, shearing and compression of brain tissues [14]. Although little is known concerning the histopathology of neonatal mild SBI in rodent models, data from repeated mild traumatic brain injury (TBI) models have provided useful information regardinPg the features of pathological progression [4, 10, 22]. Two categories of damage occur after TBI: primary damage, occurring or triggered at the moment of injury; and secondary damage, produced by complicating processes that are initiated, but do not result in apparent symptoms, at the moment of injury, and that continue to evolve for a period of time after injury. MHs and alteration of cerebral blood flow such as ischemia are involved in primary and secondary damage, respectively [6]. MHs are microscopic bleeds caused by rupture of microvessels, and extravasation of RBCs and blood plasma forming blood clots compress the surrounding parenchymal tissue as a primary injury [6]. The spatial extent of bleeds after MH could be defined from a balance between pressures in the ruptured microvessel and surrounding parenchymal tissues. Laser-induced direct rupture of cortical microvessels produced MHs of around 100 μm in diameter that compressed surrounding parenchymal tissue, but this compression was insufficient to crush nearby capillaries [19]. The mean diameter of MHs in the present model was 41.0 ± 7.7 μm in the hippocampus and 120.3 ± 21.8 μm in the cortex, suggesting that most MHs ruptured from microvessels (arterioles, venules, and capillaries). Primary neuronal damage due to MHs after neonatal SBI thus seemed very local and low.

Based on the topology of the rodent cortical angioarchitecture, 1 mm^3^ of rat cerebral cortex contains an average of 6 penetrating arterioles and 16 penetrating venules [21]. The pial arteries form part of the leptomeningeal anastomosis as a “highly redundant grid”, from which penetrating arterioles dive straight down into the cortical parenchyma [16]. In the present study, MHs in the cerebral cortex showed wedge- or column-shaped aggregations of RBC, which occasionally reached the pial surface. MHs located in the leptomeningeal anastomosis seen on P3 had disappeared for P7 rats. In the cortical and hippocampal parenchyma of P3 rats, DAB and RECA-1 double-stained sections showed that the MHs comprised of extravasated RBCs. Adjacent sections stained by DAB and iron histochemistry indicated that MHs almost entirely comprised RBCs, suggesting the H-type staining on P3–4. These areas were clearly demonstrated as hypointense areas on SWI. RBCs gradually disappeared by P6–7, indicating lysis of RBCs. Conversely, signs of iron leakage (punctate staining) and iron-uptake cells appeared around MHs (M-type staining). Because SWI enables measurement of brain iron deposition [12], hypointense signals representing leaked iron and iron-deposit cells were still observable on P6. At P12–14 in S group rats, H-type staining had almost completely disappeared, and M- and P-type stainings were evident. The present study combining MRI and histochemical analyses clearly demonstrated these transitions. Iron accumulation including punctate staining and the presence of iron-uptake cells have been known as sensitive indicators of hypoxic-ischemic injury in the brain [17]. Alteration of cerebral blood flow, especially the severity of impact of vessel occlusion is primarily correlated to hypoxic-ischemic condition in downstream regions. Severe drops in blood flow through brain tissue leads to cerebral infarction. Taken together, the present data strongly support the notion that MHs and surrounding areas are locally in an ischemic-hypoxic state. Furthermore, this model showed no prominent signs of infarctions such as edematous swelling (devoid of methyl green-stained cells) throughout the experimental period, indicating that ischemic-hypoxic state is transient. Interestingly, distance from the pial surface to the MH core in P7 differed between H- and M-types, indicating that the ischemic-hypoxic state may occur in downstream areas. No iron-staining structures were identified in the leptomeningeal plexus after P7. A high redundancy of blood flow to the leptomeningeal plexus may prevent an ischemic-hypoxic state.

In the cerebral parenchyma, both penetrating arterioles and venules form bottlenecks in the supply of neighborhood microvessels [21]. Laser-induced clotting (photothrombosis) has revealed the occlusion of penetrating vessels in the cerebral cortex results in more severely decreased capillary blood flow in the downstream circulation, indicating the local ischemia [15, 20]. However, an experiment with laser-induced MHs demonstrated that ruptured penetrating vessels still contain flow [19]. The remaining flow seems likely to contribute to a transient ischemic-hypoxic state, and further presence of iron leakage and iron-uptake cells. The present study could not determine what types of vessels ruptured in neonatal SBI, but our ongoing study is likely to solve this problem.

In addition to MHs, a study of laser-induced MHs also demonstrated that blood plasma dissipates extensively into the brain parenchyma, several hundred micrometers from ruptured vessels, and is limited by clotting [19]. Blood plasma includes several cytotoxic substances, such as thrombin, plasminogen, glutamate, and iron, causing secondary brain injury [3, 19, 26]. Since plasma iron is mostly bound to transferrin, the ruptured cerebral blood vessels and dysfunction of the blood-brain barrier (BBB) could permit extravasation of transferrin-bound iron. Thus, in the present study, punctate iron reactions may have indicated the extent of extravasation of transferrin-bound iron. Furthermore, degradation of hemoglobin following RBC lysis in the MHs produces free iron. Normally, free iron in the brain is taken up by neuronal cells (neurons, astrocytes, and microglia) via divalent metal transporter 1, and sequestered within ferritin in these cells [3]. In the present vestigial MHs, some Iba1-positive microglia and a few GFAP-positive astrocytes were found to contain iron. Intracerebral hemorrhage is known to cause iron overload and dysregulation of iron metabolism in the brain. Such dysregulation results in the accumulation of redox-active ferrous iron and oxidative stress [3, 9, 26]. Local oxidative stress was thus considered to have occurred in the present experimental model. We recently demonstrated that neonatal SBI causes persistent changes to brain activity in adults in specific regions that react to psychological stress exposure, and these changes lead to neuroendocrine and behavioral alterations [25]. Oxidative stress due to iron leakage from MHs is thus considered a primary cause of the pathophysiology in this model. Further neuropathological studies should examine the correlation between iron-induced local oxidative stress and brain activity changes in this model.

In previous experimental rat TBI models, microvascular damage occurred in white matter from 24 hr to 3 months after injury. This was associated with prolonged inflammation and BBB disruption, and delayed white matter damage causing decreased thickness of the CC [2, 5]. The present model demonstrated no significant differences in thickness of the CC between shaking and control rats at P70, indicating no delayed white matter injury in this model.

## V Conflicts of Interest

The authors declare that there are no conflicts of interest.

## VI Acknowledgement

The authors would like to thank Dr. Kawamata for assistance in measurements of stained sections and SaO_2_ analysis.
